# PARP Inhibitors in Prostate Cancer–the Preclinical Rationale and Current Clinical Development

**DOI:** 10.3390/genes10080565

**Published:** 2019-07-26

**Authors:** Verneri Virtanen, Kreetta Paunu, Johanna K. Ahlskog, Reka Varnai, Csilla Sipeky, Maria Sundvall

**Affiliations:** 1 Institute of Biomedicine, and Cancer Research Laboratories, Western Cancer Centre FICAN West, University of Turku, FI-20520 Turku, Finland; 2 Faculty of Science and Engineering, Åbo Akademi University, and Turku Bioscience, University of Turku and Åbo Akademi University, FI-20520 Turku, Finland; 3 Department of Primary Health Care, University of Pécs, H-7623 Pécs, Hungary; 4 Faculty of Health Sciences, Doctoral School of Health Sciences, University of Pécs, H-7621 Pécs, Hungary; 5 Institute of Biomedicine, University of Turku, FI-20520 Turku, Finland; 6 Department of Oncology and Radiotherapy, Turku University Hospital, FI-20521 Turku, Finland

**Keywords:** castration resistant prostate cancer, DNA damage repair, PARP inhibitors, precision medicine

## Abstract

Prostate cancer is globally the second most commonly diagnosed cancer type in men. Recent studies suggest that mutations in DNA repair genes are associated with aggressive forms of prostate cancer and castration resistance. Prostate cancer with DNA repair defects may be vulnerable to therapeutic targeting by Poly(ADP-ribose) polymerase (PARP) inhibitors. PARP enzymes modify target proteins with ADP-ribose in a process called PARylation and are in particular involved in single strand break repair. The rationale behind the clinical trials that led to the current use of PARP inhibitors to treat cancer was to target the dependence of BRCA-mutant cancer cells on the PARP-associated repair pathway due to deficiency in homologous recombination. However, recent studies have proposed therapeutic potential for PARP inhibitors in tumors with a variety of vulnerabilities generating dependence on PARP beyond the synthetic lethal targeting of BRCA1/BRCA2 mutated tumors, suggesting a wider potential than initially thought. Importantly, PARP-associated DNA repair pathways are also closely connected to androgen receptor (AR) signaling, which is a key regulator of tumor growth and a central therapeutic target in prostate cancer. In this review, we provide an extensive overview of published and ongoing trials exploring PARP inhibitors in treatment of prostate cancer and discuss the underlying biology. Several clinical trials are currently studying PARP inhibitor mono-and combination therapies in the treatment of prostate cancer. Integration of drugs targeting DNA repair pathways in prostate cancer treatment modalities allows developing of more personalized care taking also into account the genetic makeup of individual tumors.

## 1. Introduction

Although advances have been made in early detection and treatment of localized prostate cancer, metastatic prostate cancer is one of the most common causes of male cancer deaths. The standard first line treatment of metastatic prostate cancer is androgen deprivation therapy (ADT). ADT can be combined with docetaxel chemotherapeutic agent or androgen receptor (AR) signaling inhibitors at first line setting or later when castration resistance has developed [1]. However, the majority of patients eventually develop a resistant disease, which does not respond to any current therapies. New clinical targets and therapies are needed to develop better and more personalized treatment options for aggressive and castration resistant prostate cancer (CRPC). In this review, we discuss the significance of targeting DNA damage repair pathways in prostate cancer and focus in particular on covering the biological background and clinical potential of drugs inhibiting Poly(ADP-ribose) polymerase (PARP) enzymes which are also uniquely connected to AR signaling.

## 2. DNA Damage Response and PARP

In the course of evolution, a complex signaling pathway known as DNA damage response (DDR) developed to handle endogenous threats arising during cellular metabolism and hydrolytic reactions or exogenous insults constantly damaging the DNA. Maintaining the genomic integrity is needed for preventing diseases, such as cancer, which are associated with genomic instability. Genomic instability is an inherent property of most malignancies [2]. In order for cells to proliferate they proceed through cell cycle by entering checkpoints in which they must pass certain signaling requirements. During checkpoints, protein complexes assess the condition of cellular DNA and react on aberrations. DDR is a complicated system with three phases–detection, accumulation of repair factors and physical repair. Within DDR simultaneous pathways exist that can accommodate shortcomings of parallel mechanisms. DDR monitors integrity of the DNA and in the case of lesion, it activates cell cycle arrest and DNA repair machinery [3].

Poly(ADP-ribose) polymerase (PARP) family has a significant role in DNA repair. PARP protein family is composed of 17 members, but however, only PARP1 and PARP2 are related to DDR [4] PARP1 is a highly conserved multifunctional enzyme and it binds to DNA breaks and recruits DNA repair proteins to the damaged site. It is involved in regulation of chromatin structure and DNA metabolism, and it is a survival factor that plays a role in the maintenance of genomic integrity [5,6,7]. It has been estimated that the catalytic activity of PARP1 is stimulated 500 fold by single strand or double strand DNA breaks [4]. Upon detection of a DNA strand break, PARP1 binds to the DNA through its DNA-binding domain, cleaves nicotinamide adenine dinucleotide (NAD+), and then transfers the resulting ADP-ribose onto itself or other target proteins by formation of a covalent bond between the protein and the ADP-ribose or subsequent polymers of ADP-ribose [8,9]. This reaction called PolyADP-ribosylation (PARylation), is a post-translational modification that auto-activates PARP and acts as a signal for other DNA-repairing enzymes and DNA repair [4,8].

**Figure 1:**
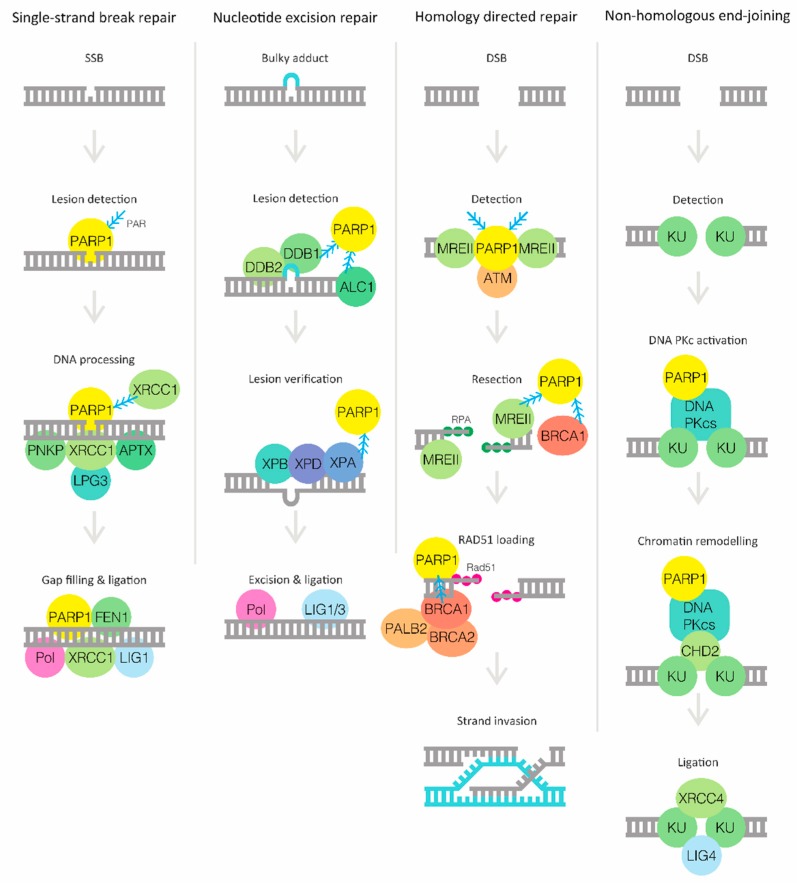
Basic principles and components of major DNA damage response (DDR) pathways in which Poly(ADP-ribose) polymerase 1 (PARP1) has a fundamental role. PARP1 detects the DNA lesion in single strand break repair (SSBR) and in homologous recombination (HR), which is the most common form of homology directed repair [11,20]. PARP1 generally takes part in recruiting repair factors to the lesion site and later interacts with or promotes activity of enzymes during physical repair stage of DDR [12,13,14,15,26].

DNA damage may occur as single strand breaks (SSB) or double strand breaks (DSB) in response to a variety of DNA damaging agents and conditions. The most common type of DNA damage are single base modifications and nucleotide damage caused by reactive endogenous metabolites and by spontaneous base modifications, such as oxidations of bases or generation of abasic sites [10]. This type of damage is repaired by excision repair mechanisms, including single strand break repair (SSBR), base excision repair (BER) and nucleotide excision repair (NER) (Figure 1). PARP1 is essential especially in SSBR by detecting and binding to SSBs followed by addition of PAR to PARP1 itself leading to its activation [11]. Active PARP1 then recruits X-ray repair cross-complementing protein 1 (XRCC1), a core repair factor that acts as a scaffold for polynucleotide kinase 3’-phosphatase (PNKP), aparataxin (APTX) and DNA ligase 3 (LIG3) to process the SSB. This is followed by a gap filling step carried out by DNA polymerase δ (Pol δ), Pol ε and Pol β, in addition to PARP1 that promotes the activity of flap endonuclease 1 (FEN1). The final DNA ligation is performed by LIG1 [12].

The role of PARP1 in BER is still unclear; however, PARP1 plays an important role in NER during initial steps of damage recognition as well as during recruitment of subsequent repair proteins (Figure 1). The DNA-damage-binding protein 1 (DDB1)-DDB2 complex is recruited to autoPARylated PARP1 at the DNA lesion, followed by the recruitment of amplified in liver cancer protein 1 (ALC1) resulting in chromatin decondensation [13,14]. This is followed by recruitment of xeroderma pigmentosum group C-complementing protein (XPC) and RAD23B leading to lesion verification involving binding of XPB and XPD through interaction with XPA and PARP1. Finally, the damaged site is excised by nucleases, excision repair cross-complementing group 1 protein (ERCC1) and XPG, and the gap is filled by Pol δ, Pol ε and Pol κ, and ligated by LIG1 and LIG3 [15].

DSBs are formed upon exposure to DNA damaging agents, such as ionizing irradiation, endogenously during programmed genome arrangements or from replication fork collapse [16]. The two main DSB repair pathways in eukaryotic cells are homologous recombination (HR) and non-homologous end joining (NHEJ) (Figure 1) [17]. HR requires a DNA template for error-free repair and occurs therefore after replication in S and G2 phases of the cell cycle while the error-prone NHEJ is the predominant repair pathway during G1 phase. In addition to detecting single strand breaks, PARP1 also recognizes DSBs [18]. The key kinases involved in the repair of DSBs belong mainly to phosphatidylinositol 3-kinase-like protein kinase (PIKKs) family [3]. These proteins include atypical serine-threonine kinases ataxia telangiectasia mutated (ATM), ataxia telangiectasia and Rad3 related (ATR) and DNA-dependent protein kinases (DNA-PKs) [19]. After detecting and binding to a DSB, PARylated PARP1 promotes recruitment of ATM and the Mre11-Rad50-Nbs1 (MRN) complex through binding of meiotic recombination 11 (Mre11) to activate HR [20]. While ATM activates checkpoint and repair pathway proteins by phosphorylation, the MRN complex initiates DNA end resection to enable binding of replication protein A (RPA) to the resulting single stranded DNA. For the next step in repairing the lesion, PARP1 is involved in recruiting breast cancer type 1 susceptibility protein (BRCA1) to the damaged site in complex with BRCA2 and partner and localizer of BRCA2 (PALB2) [21]. BRCA2 delivers RAD51 to the damaged site, which is required to initiate strand invasion and finally repair through D-loop formation and DNA synthesis [22,23,24]. Chk1 and Chk2 are serine-threonine kinases acting downstream of ATM and ATR that facilitate the DNA damage response by initiating cell cycle arrest thereby allowing time for repair. Chk1 is phosphorylated by ATR, primarily in response to replication stress, while ATM activates Chk2 following double strand breaks. Both reactions lead to phosphorylation of CDC25 resulting in its degradation preventing release from cell cycle checkpoints [25]. When repair by NHEJ is preferred, the DSB is bound by KU70-KU80 dimers, which activate DNA-PKcs. PARP1 interacts with DNA-PK, stimulating its activity, and is also involved in subsequent steps in the repair process by facilitating the recruitment of chromodomain helicase DNA-binding protein 2 (CHD2) [14,26]. CHD2 is crucial in recruiting XRCC4 and LIG4 to the site for final DNA ligation [12].

## 3. Targeting DNA Repair Defects in Cancer with PARP Inhibitors

The development of PARP inhibitors is mainly based on two approaches: in the first strategy PARP inhibition is combined with DNA damaging therapy; and in the second, the aim is to target cells that are already genetically predisposed to die in the absence of PARP activity [27]. In 2005, two benchmark studies proved that *BRCA1* or *BRCA2* DNA repair defect causing mutations sensitize cells to PARP inhibition, which results in chromosomal instability, cell cycle arrest and subsequent apoptosis [28,29]. Mechanistically, a cell with defects in HR becomes reliant on an alternative DDR system that uses PARP as a catalyst for single stranded DNA repair (Figure 1). A PARP system allows cells with HR deficiency to stay viable but at the same time creates a pharmaceutical target as cells that lose both HR and PARP-assisted SSBR face cell death through synthetic lethality. In the synthetic lethality concept combined effects lead to cell death when present simultaneously. In the pioneering clinical trials PARP inhibitors (PARPi) were used to treat HR deficient patients with germline BRCA1/BRCA2 mutations (gBRCA1/2m) [30]. Remarkable recent results in newly diagnosed ovarian cancer patients with gBRCAm further support the proof-of-concept for synthetic lethal targeting of BRCAm patients with PARPi [31].

**Figure 2:**
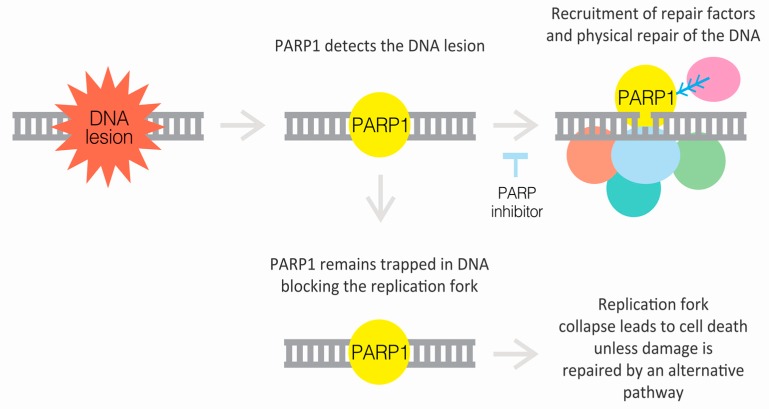
PARP inhibitors (PARPi) reduce the catalytic activity of PARPs. In addition, PARPi trap PARP at the site of DNA damage by preventing PARP from detaching from DNA. Cytotoxic PARP-DNA complexes prevent replication fork from progressing and lead to cell death unless damage is repaired [32,36].

There are two different aspects to PARP inhibitor mechanism of action–the inhibitions of catalysis and trapping of PARP enzymes at the site of DNA damage (Figure 2) [32]. According to current knowledge, all PARPi suppress the catalytic activity of both PARP1 and PARP2 [27]. This prevents the NAD^+^ binding and eventually inhibits the attachment of ADP-ribose polymers on targets [33]. Without PARylation cells cannot progress through G2 phase and enter M phase [34]. In addition, PARP inhibition has been linked to increased rates in apoptosis [35]. PARPi also traps both PARP1 and PARP2 at the site of SSB, preventing PARP from detaching from DNA. These PARP-DNA complexes are more cytotoxic than SSBs caused by deficient PARP enzymes [36]. Both PARP1 and PARP2 are parts of larger protein complexes and the meeting of the ongoing replication fork with the PARP-DNA complex eventually leads to double strand ends and replication fork collapse [32,37,38]. The potency of trapping PARP enzymes differs significantly between inhibitors. Talazoparib is more potent than niraparib, which is more potent than olaparib and rucaparib, which again are more potent than veliparib when considering the ability to trap PARP at the site of DNA damage (trapping efficiency: talazoparib >> niraparib > olaparib = rucaparib >> veliparib) [36,39]. Although some PARP inhibitors can equally efficiently target both PARP1 and PARP2, PARP1 may be the major target for them in human cells, since the expression levels of PARP1 are much higher than PARP2 [4,40].

## 4. Germline Mutations in DNA Damage Repair Genes in Prostate cancer

Multiple studies have reported association of frequent germline deleterious mutations in DDR genes with advanced prostate cancer, which defines the basis for the use of PARP inhibitors to treat prostate cancer. Specifically, germline *BRCA1/2* mutations associate with increased risk and with more aggressive prostate cancer (Gleason ≥ 8), higher risk of nodal involvement, and distant metastasis at diagnosis [41]. Subgroup analyses confirmed the poor outcomes in particular in *BRCA2* patients, whereas the role of *BRCA1* was not well defined due to the limited size and follow-up in this subgroup [41]. This was strengthened by Leongamornlert et al., who described frequent germline deleterious mutations in DNA repair genes and discovered 14 new loss-of-function (LOF) mutations in 7.3% of familial prostate cancer patients. These germline mutations were more frequently associated with nodal involvement, metastasis or T4 tumor stage [42].

In a prospective multicenter cohort study, the prevalence of germline mutations were screened in 107 DDR-associated genes. 16.2% of patients were found to be carriers (3.3% *BRCA2*, 1.9% *ATM*, 0.96% *BRCA1*). Although the impact of *ATM/BRCA1/BRCA2* germline mutations on cause-specific survival (CSS) was not statistically significant, the CSS was halved in germline *BRCA2* carriers. Germline *BRCA2* mutations were identified as an independent prognostic factor for CSS. It was concluded that germline *BRCA2* mutations have a deleterious impact on metastatic CRPC outcomes that may be affected by the first line of treatment used [43].

With respect to germline mutations in DDR genes, a recent study found the incidence of inherited DNA repair gene alterations in metastatic prostate cancer to be significantly higher (11.8%) than in men with localized prostate cancer (4.6%) and in the general population (2.7%) [44] Specifically, mutations in *BRCA2* (5.3%), *CHEK2* (1.9%), *ATM* (1.6%), *BRCA1* (0.9%), *PALB2* (0.4%), *RAD51D* (0.4%), were significantly enriched in patients with metastatic prostate cancer compared to the general population. These findings suggest that this subset of men with germline mutations in DDR genes are more likely to develop metastatic prostate cancer and may potentially benefit from PARPi therapy [44].

Prevalence of germline *BRCA2* mutations with known pathogenic annotation was reported to be significantly higher in men with advanced and metastatic prostate cancer (3.1%) compared to organ-confined disease (0.7%) [45] Racial variation was also observed: African American patients carried more frequently *BRCA1/2* variants of unknown significance (VUS) when compared to Caucasian Americans (4.6 vs. 1.6%, respectively). However, the prevalence of pathogenic mutations was similar across the races.

## 5. Somatic Mutations in DNA Damage Repair Genes in Prostate Cancer

Multiple candidate driver mutations in DDR and collaborating AR signaling pathway genes have been identified through exome sequencing of lethal, heavily pre-treated mCRPC autopsy samples [46]. Two deleterious somatic mutations in PRKDC (I1137 frame shift and E640 non-sense) encoding the catalytic subunit of the DNA-PK were reported in a patient with extremely aggressive localized prostate cancer. In addition, high level focal deletions of genes involved in DNA repair in hypermutated CRPC samples were found. For example, somatic, focal homozygous deletion in the mismatch repair gene MSH2, and a somatic homozygous deletion of a ~2 MB region on chr13 harboring BRCA2 [46].

Further discoveries of genomic landscape of mCRPC showed that approximately 23% of patients harbor somatic DNA repair pathway aberrations [47]. Of these, *BRCA2*, *BRCA1*, and *ATM* account for 19.3% overall and they were substantially more frequent in mCRPC compared to those in primary prostate cancers. 12.7% of cases had loss of *BRCA2*, of which 90% exhibited biallelic loss. Of note, 5.3% of mCRPC patients harbored pathogenic germline *BRCA2* mutations with a subsequent somatic event that resulted in biallelic loss, revealing a high frequency relative to primary prostate cancer. In addition, mutational events were noted in *CDK12, FANCA, RAD51B* and *RAD51C* [47]. Also other studies have showed similar results finding approximately 12% and 8% of prostate cancer patients carrying a BRCA2 or ATM mutation, respectively [48]. Somatic *BRCA2* mutations have been suggested to arise early in tumors from patients who eventually develop metastatic disease, while *ATM* alterations seem to enrich in CRPC [49].

## 6. Crosstalk between AR Signaling and DNA Damage Response

AR signaling is one of the major growth promoting pathways in prostate cancer, and ADT is the corner stone of prostate cancer treatment. Several lines of evidence suggest that prostate tumor cells are uniquely connected to DDR through AR signaling. AR signaling inhibitors seem to down-regulate DDR gene expression and increase DNA damage [50]. ADT results in the state of BRCAness, a phenotype with treatment susceptibility analogous to that of BRCA dysfunctional cancer, including HR deficiency and increased PARP activation, leading to sensitivity of prostate cancer to PARP inhibition in combination with AR signaling inhibitors in preclinical setting [51]. In prostate cancer xenograft experiments, a superior effect was observed when enzalutamide was administered prior to olaparib when compared to olaparib or enzalutamide monotherapy [52]. PARP1 expression is significantly up-regulated in several cancers including breast and ovarian cancer, although up-regulation is less striking in prostate cancer [53]. Moreover, PARP may promote the transcriptional activity of AR in prostate cancer [54]. Thus, PARPs may have a role as drug targets in prostate cancer beyond being targeted as a part of the concept of synthetic lethality in tumors with DDR gene mutations. Increased PARP1 activity correlates also with more advanced disease and poor outcome in prostate cancer [55]. However, changes in PARP1 activity appear to be unaffected by DSBs or increased PARP1 expression [55]. In addition, in genetic mouse models, loss of PARP activity has been linked to increase prostate tumorigenesis, suggesting that the role of PARP in prostate cancer is complex [56]. Interestingly, also combination of AR signaling inhibitors with inhibitors of Chk1 promotes DNA damage and eventually leads to cell death, supporting the close relationship between AR signaling and DDR [57].

## 7. Clinical Development of PARP Inhibitors in Prostate Cancer

Four PARP inhibitors currently have an FDA approval and an indication in treating ovarian and breast cancer (Table 1). A few early phase studies have already been completed in the treatment of prostate cancer with PARP inhibitors (Table 2). Several ongoing clinical trials are currently studying different PARP inhibitors as monotherapy or in combination with other treatments ranging from chemotherapy, AR signaling inhibitors, radical prostatectomy, radiation therapy, immune therapy and different targeted agents (Table 3).

A phase II study evaluating the efficacy of olaparib for preselected gBRCA1m or gBRCA2m solid tumors showed 50% tumor response rates in CRPC patients [60] In a phase II TOPARP study, treatment with olaparib in CRPC patients with germline or somatic DDR gene defect also led to a high response rate (88%). Homozygous deletions, deleterious mutations, or both in DDR genes, including *BRCA1/2, ATM*, *CHEK2*, and *PALB2*, were detected in 33% of mCRPC patients enrolled in the study. Importantly, all patients with *BRCA2* loss, and 4 of 5 patients with *ATM* aberrations, had a response to olaparib [61] Only a few responses (6 %) were detected in patients without DDR gene mutations [69] The ongoing phase 3 trials will give further insight if patients with DDR mutations benefit from PARPi. In the PROfound phase 3 study (NCT02987543) evaluating the efficacy of olaparib vs. physicians choice of either entzalutamide or abiraterone in metastatic CRPC, patients that are included should have an aberration in one of 15 genes analyzed by an HR repair assay (Foundation Medicine, Inc., Cambridge, MA, USA). Cohort A (n = 240 approx.) includes patients with mutations in *BRCA1*, *BRCA2* or *ATM*, while patients with a mutation in 12 other HR genes will be assigned to Cohort B (n = 100 approx) [70] TRITON3 is an ongoing trial (NCT02975934) evaluating rucaparib monotherapy vs. physicians choice of either enzalutamide, abiraterone or docetaxel in patients with metastatic CRPC and a deleterious germline or somatic *BRCA1*, *BRCA2*, or *ATM* mutation identified by prior local testing or central testing during screening [71].

AR pathway’s distinct role in CRPC and especially its linkage to DDR explored in preclinical trials suggests unique opportunities for combining PARPi with AR signaling inhibitors and castration [72]. The efficacy of olaparib in combination with abiraterone was assessed in a randomized, placebo-controlled phase II study [62]. Significantly longer radiographic progression-free survival (rPFS) was reported in favor of olaparib plus abiraterone (13.8 vs 8.2 months) [62]. Surprisingly, rPFS was significantly better in favor of olaparib arm in patient subpopulation without HR defect and no evidence suggested that DDR gene mutations would sensitize to PARPi in this context [62]. Veliparib plus abiraterone combination showed also promising efficacy (Table 2) [68]. Patients were selected based on ETS transcription factor gene fusions, which did not predict response. However, patients with biallelic DDR mutations had significantly more responses and better median PFS [68]. The ongoing PROPEL phase 3 study (NCT03732820) is investigating olaparib in the combination with abiraterone in the first line setting for metastatic CRPC without prescreening patients for HR aberrations [73]. Talapro-2 in turn is an ongoing 2-part phase 3 trial (NCT03395197) analyzing the efficacy of the combination of enzalutamide plus talazoparib vs. enzalutamide plus placebo in both unselected patients and in patients with DDR mutations [74]. In the phase 3 MAGNITUDE study (NCT03748641) comparing abiraterone plus placebo to abiraterone plus niraparib during the prescreening phase participants will be evaluated for DDR gene defects identified by the sponsor’s required assays and then will be assigned to one of the two cohorts, the other containing only patients tested negative for and the other positive for DDR deficiency [75].

PARPi has also been studied in combination with DNA damaging chemotherapy. According to one phase I study, veliparib was well tolerated in combination with temozolomide but combination had only modest efficacy in CRPC [65]. One ongoing phase II study is evaluating the efficacy of PARPi in combination with docetaxel and carboplatin (Table 3).

Several lines of preclinical evidence suggest synergy between PARPi and immune checkpoint inhibitors. For example, double strand break repair pathway has been suggested to regulate PD-L1 expression in cancer [76]. Interestingly, PARP inhibition has also been shown to increase PD-L1 expression in cells depleted of BRCA2 [76]. Several checkpoint inhibitor therapy targeting immune checkpoints, including PD1 blockers nivolumab, pembrolizumab and JNJ-63723283, and PDL1 blockers avelumab and durvalumab, are combined with PARPi in the current trials in prostate cancer (Table 3). Interestingly, olaparib plus durvalumab combination demonstrated efficacy in prostate cancer patients with DDR mutations in an early phase study (Table 2) [63]. An ongoing phase 3 trial KEYLYNK-010 (NCT03834519) evaluates olaparib in the combination with pembrolizumab vs. abiraterone and prednisolone or enzalutamide in genetically unselected patients [77].

Cell signaling pathways such as PI3K-Akt pathway have been implicated in regulation of BRCAness and sensitivity to PARPi [78,79]. Preclinical evidence suggests efficacy for the combination of PARPi and Pi3K pathway inhibition in prostate cancer [80]. PARP inhibitors are currently explored in the ongoing trials also in prostate cancer in the combination with drugs targeting cell signaling pathways, such as an AKT inhibitor ipatasertib, and a VEGFR inhibitor cediranib. Moreover, PARPi are evaluated in the combination with other DDR components than PARP, such as ATR inhibitor AZD6738 (Table 3).

### Adverse Events and Tolerability

PARPi are in general relatively well tolerated as monotherapy, and toxicity can often be managed with dose reductions. Typical adverse effects are myelotoxicity, in particularly anemia, fatigue and gastrointestinal symptoms [61]. However, combining PARPi with other drugs may increase likelihood of toxicity. For example, in a phase II trial of PARPi combined with abiraterone typical grade 1–2 adverse events included nausea, constipation and fatigue, and grade 3 included anemia [62]. Significantly more serious adverse events were reported in patients treated with olaparib and abiraterone (34%) when compared to abiraterone alone (18%), including myocardial infarctions and one treatment-related death due to pneumonitis [62]. Veliparib plus abiraterone combination was relatively well tolerated [68]. 24% of patients (n = 19) had grade 3 treatment-related adverse effects compared to 20% in abiraterone alone arm. One patient had grade 4 thrombocytopenia, and one patient had grade 5 cardiac arrest possibly treatment-related in veliparib plus abiraterone arm [68]. In a phase I trial of PARPi combined with temozolomide (n = 26) serious treatment–emergent adverse effects were reported in 26.9% subjects, including colitis (n = 2), hepatorenal syndrome, hyperglycemia, bone pain, mental status change, hematuria, urinary tract obstruction, epistaxis and deep vein thrombosis (all n = 1) [65]. Olaparib plus durvalumab showed acceptable toxicity profile in an early phase study [63]. The ongoing phase 3 trials will give further information regarding tolerability in prostate cancer patients in different settings (Table 3).

## 8. Predictive Markers of Response to PARP Inhibitors

Initial studies showed that HR deficiency due to BRCAm predicts response to PARPi [28,29,30]. Preclinical evidence suggests that tumors with mutations in other DDR genes, such as ATM, and HR regulators are also sensitive to PARPi [81,82,83]. Several potential predictive markers of sensitivity have been suggested in preclinical setting, including phosphatases and several kinases regulating DNA damage response and cell cycle [84,85]. In prostate cancer, high response rates have been reported in tumors carrying either germline or somatic mutations in DDR genes [60,61]. However, tumors with BRCA mutations appear to be more sensitive to PARPi olaparib than tumors with ATM mutations in CRPC [86]. Since the functional consequence of a mutation is not always known and the factors resulting in HR deficiency are complex, it is important to assess the functional status of HR in patient samples. Functional HR assays, including ex vivo RAD51 foci assays, have been proven beneficial and have additional value to gene sequencing in identifying patients that may benefit of PARPi in breast and ovarian cancer [87,88]. The ongoing phase 3 trials are expected to shed more light on the validity of the synthetic lethality concept in prostate cancer context.

Responses to PARPi have been detected in combination with androgen receptor inhibitors in prostate cancer also in patients without obvious mutations causing HR defect in tumors [62], suggesting that blocking AR signaling may induce clinically relevant state of BRCAness to tumors [51]. Hypoxic tumors in particular are suggested to be sensitive to PARPi [89].

## 9. Mechanisms of Intrinsic and Acquired Resistance to PARP Inhibitors

The major clinically described form of PARPi resistance is restoration of HR competency by a reversing secondary mutation of a HR gene [90]. For example, sequencing cell-free DNA from ovarian carcinoma patients treated with rucaparib identified BRCA reversion mutations in gBRCAm and somatic BRCAm carriers, and reversion mutations eventually associated with disease progression [91]. A HR–independent mechanism protecting the stalled replication forks can sustain viability in HR deficient cells in presence of PARPi. It is proposed that if cells survive the initial BRCA-null crisis because the replication forks are transiently protected by a defect in MRE11 recruitment, their survival is no longer dependent of PARP inhibition. Loss of PTIP or CHD4 have been observed to associate fork protection with resistance to PARPi [92,93]. In prostate cancer patients, BRCA2 reversion mutations have been associated with PARPi resistance [94]. Loss of factors regulating DSB end resection can cause PARPi resistance. In BRCA1 mutated cell lines, 53BP1 suppresses resection in HR, promoting NHEJ instead. Loss of 53BP1 function allows HR to occur and 53BP1 loss has been shown to confer resistance to PARPi [95,96]. Deletion of RIF1, a factor cooperating with 53BP1 in 5’ end resection, reduced cytotoxicity of PARPi in mice [97]. Downstream of 53BP1, REV7 was also found to coordinate repair pathway choices and to affect PARPi resistance in preclinical experiments [98]. Similarly loss of CST complex, functioning as a resection antagonist, leads to restoration of HR independently of BRCA1, causing PARPi resistance [99]. Poly(ADP-ribose) glycohydrolase (PARG) is an enzyme capable of removing PAR chains. PARG suppression was found to partially restore PARP1 signaling in the presence of PARPi in mouse *Brca2* mutant cell lines and organoids [100]. Sensitivity and resistance mechanisms in combination therapy settings of prostate cancer are likely more complex due to signaling cross talk or other factors that can affect HR competency, as suggested in combination therapy trial with abiraterone and olaparib [62]. Using in vitro and mouse xenograft models *Dréan* et al. established that exposing tumor cell populations to platinum salt or PARPi therapy produces secondary mutated clones in a Darwinian fashion. Created PARP resistant tumor populations were; however, found to be sensitive to a WEE1 kinase inhibitor AZD-1775, encouraging the possibility of subsequent treatment options after acquired resistance [101].

## 10. Future Perspectives

During recent years, advances have been made in understanding the underlying genetic events and biology in different steps of prostate cancer development, but yet no genetic subtypes have been established with prognostic and/or predictive value for clinical use. Recent data suggests that high proportion of prostate cancer patients carry mutations in DNA damage repair genes. These patients may represent a new subgroup of prostate cancer patients that benefit from therapeutics targeting DNA damage response pathways, such as PARP inhibitors. DNA damage response involves hundreds of proteins and many of them are currently investigated as drug targets, in addition to PARPs [102]. Many of these emerging DDR modifiers being developed, such as DNA-PK inhibitors studied preclinically, have promising potential in prostate cancer [103,104]. This opens opportunities to develop more personalized therapeutic modalities for prostate cancer patients taking into account their tumor genomes in the future. However, deeper understanding of DDR biology in prostate cancer context and in conjunction with AR signaling is needed to fully exploit the potential of DDR targeting drugs for the benefit of patients. The first currently ongoing large phase 3 trials will tell if PARP inhibitors enter to the treatment options for CRPC in the near future. More work is defeinitely needed to understand what the best combinations and treatment modalities with PARPi might be.

## Figures and Tables

**Table 1:** PARP inhibitor drugs approved by the FDA.

Compound	Company	Indications	Date of Approval	Stage of Development for PCa
Olaparib (Lynparza ^®^)	AstraZeneca Pharmaceuticals LP (Cambridge, UK)	gBRCA-mutated advanced ovarian cancer	December 2014	III
		Maintenance treatment of recurrent epithelial ovarian, fallopian tube or primary peritoneal cancer	August 2017	
		gBRCA-mutated HER2-negative metastatic breast cancer	January 2018	
		Maintenance treatment of gBRCA- or sBRCA-mutated advanced epithelial ovarian, fallopian tube or primary peritoneal cancer	December 2018	
Rucaparib (Rubraca ^®^)	Clovis Oncology, Inc. (Boulder, CO, USA)	gBRCA- or sBRCA-mutated advanced ovarian cancer	December 2016	III
		Maintenance treatment of recurrent epithelial ovarian, fallopian tube, or primary peritoneal cancer	April 2018	
Niraparib (Zejula ^®^)	Tesaro, Inc., (Waltham, MA, USA)	Maintenance treatment of recurrent epithelial ovarian, fallopian tube, or primary peritoneal cancer.	March 2017	III
Talazoparib (Talzenna ^®^)	Pfizer, Inc. (New York, NY, USA)	gBRCA-mutated HER2-negative locally advanced or metastatic breast cancer	October 2018	III

Pca: Prostate cancer; germline BRCA (gBRCA) or somatic BRCA (sBRCA) mutation.

**Table 2:** Efficacy results of PARP inhibitor treatment in prostate cancer.

Treatment Regimen	Phase	Number of Pca Patients	Biomarkers	CR (%)	PR (%)	SD (%)	PD (%)	OS (Months)	PFS (Months)	Ref.
Niraparib	I	21	BRCAm 5 % (n = 1)	0		43				[58]
Olaparib	I	3	BRCA2m 33 % (n = 1)							[30]
Olaparib or niraparib	I	4	BRCA2m 100 % (n = 4)	0	25	25	50			[59]
Olaparib	II	8	gBRCA2m 87.5% (n = 7), gBRCA1m 12.5 % (n = 1)	0	50	25	25	18.4	7.2	[60]
Olaparib (TOPARP-A)	II	50	Overall 33 % (n = 16), BRCA2m (n = 7), ATMm (n = 5), BRCA1m or CHEK2m with FANCAm (n = 3), PALB2m (n =1), HDAC2m (n = 1)	0	19	6		13.8 vs 7.5 *	9.8 vs 2.7 *	[61]
Abiraterone and prednisolone with or without olaparib	II	71 vs 71 †	HRR mutation 15 vs 14 % †	0	27 vs 32 †, ns	48 vs 21 †	21vs 47 †	22.7 vs 20.9 †, ns	13.8 vs 8.2 †	[62]
Olaparib and durvalumab	II	17	DDRm 77% of responders‡, gBRCA2m 33%, sBRCA2m 22%, gNBNm 11%, PMS2m 11%		24				16.1	[63]
Talazoparib	I	4								[64]
Veliparib and temozolomide	I	26		0	0§			9.2	2.1	[65]
Veliparib	I	3	BRCA2m 100%	0	66	33				[66]
Veliparib, carboplatin and gemcitabine	I	1	BRCAm 100 % (n = 1)	100						[67]
Abiraterone and prednisolone with or without veliparib	II	79 vs 74 †	Overall DDRm 31% (n = 25) of 80 analyzed, BRCA2m (n = 15), ATMm (n = 4), BRCA1m (n = 4), RAD51Bm (n = 1), RAD51Cm (n = 1), PALB2m (n = 1), FANCAm (n =1)	0 vs 3 †	52 vs 45 †	26 vs 35 †	17 vs 20 †	32.3 vs 30.6 †	11 vs 10.1 †	[68]

* Biomarker-positive group vs biomarker-negative group. † PARPi vs control. ‡ Subjects with PSA decline of ≥50%. §None of the measurable disease achieved an objective response according to RECIST. CR: Complete response; HRR: Homologous recombination repair; OS: Overall survival;; PFS: Progression-free survival; PD: Progressive disease; PR: Partial response; SD: Stable disease; ns= not significant. Information was compiled by searching the Pubmed and Web of Science databases. The search terms included ‘Prostate cancer’, ‘Olaparib’, ‘AZD2281’, ‘KU-0059436’, ‘Rucaparib’, ‘Niraparib’, ‘MK-4827’, ‘Talazoparib’, ‘BMN 637’, ‘MDV3800’, ‘Veliparib’, ‘CEP-9722’, ‘Pamiparib’ and ‘BGB-290’.

**Table 3:** Ongoing clinical trials using PARP inhibitors to treat prostate cancer.

Treatment Regimen	Status	Allocation	HRD Selection	Estimated Enrollment	Phase	CTID
**PARPi monotherapies**						
Niraparib	Recruiting		Yes	301	II	NCT02854436
Olaparib	Recruiting		No	89	II	NCT01682772
Olaparib	Active, not recruiting	Randomized	Yes	340	III	NCT02987543
Olaparib	Recruiting		Yes *	50	II	NCT03047135
Olaparib	Recruiting	Randomized	No	96	II	NCT03263650
Olaparib	Recruiting		Yes	27	II	NCT03434158
Pamiparib	Recruiting		Yes	100	II	NCT03712930
Rucaparib	Recruiting		Yes	360	II	NCT02952534
Rucaparib	Recruiting		Yes	30	II	NCT03413995
Rucaparib	Recruiting		Yes	29	II	NCT03533946
Talazoparib	Recruiting		Yes	100	II	NCT03148795
**PARPi + AR signaling inhibitors**						
Niraparib and Abiraterone and Prednisolone	Recruiting	Randomized	Yes	1000	III	NCT03748641
Olaparib or Olaparib and Abiraterone and Prednisone	Recruiting	Randomized	Yes	70	II	NCT03012321
Olaparib and Abiraterone and Prednisolone	Recruiting	Randomized	No	720	III	NCT03732820
Rucaparib and Abiraterone, Enzalutamide or Docetaxel	Recruiting	Randomized	Yes	400	III	NCT02975934
Niraparib and Apalutamide or Abiraterone and Prednisolone	Active, not recruiting		No	34	I	NCT02924766
Niraparib and Enzalutamide	Terminated (Suspended by funder)		No	2	I	NCT02500901
Talazoparib and Enzalutamide	Recruiting	Randomized	Yes†	872	III	NCT03395197
**PARPi + immune checkpoint inhibitors**						
Talazoparib and Avelumab	Recruiting	Non-Randomized	No	242	Ib/II	NCT03330405
Olaparib and Durvalumab	Recruiting		Yes	32	II	NCT03810105
Niraparib and JNJ-63723283 or Abiraterone and Prednisolone	Recruiting	Non-Randomized	Yes	150	Ib–II	NCT03431350
Rucaparib and Nivolumab	Recruiting	Non-Randomized	No	330	II	NCT03338790
Rucaparib or Rucaparib and Nivolumab	Recruiting	Randomized	No	60	Ib/Iia	NCT03572478
Olaparib and Pembrolizumab	Recruiting	Non-Randomized	No	400	I	NCT02861573
Olaparib and Pembrolizumab	Not yet recruiting	Randomized	No	780	III	NCT03834519
**PARPi + chemotherapy agents**						
Rucaparib, Docetaxel and Carboplatin	Recruiting		Yes	20	II	NCT03442556
Pamiparib and Temozolomide	Recruiting	Non-Randomized	Yes	150	I	NCT03150810
**PARPi + radionuclide therapies**						
Niraparib and Radium Ra 223 Dichloride	Recruiting		No	6	I	NCT03076203
Olaparib and Radium Ra 223 Dichloride	Recruiting	Randomized	No	112	II	NCT03317392
Olaparib and 177Lu-PSMA	Not yet recruiting		No	52	I	NCT03874884
**PARPi + surgical procedures**						
Olaparib and RP	Recruiting		Yes	13	II	NCT03432897
Olaparib and RP	Recruiting		Yes	15	II	NCT03570476
**PARPi + VEGF RTK inhibitors**						
Olaparib and Cediranib	Active, not recruiting	Randomized	No	90	II	NCT02893917
**PARPi + AKT inhibitors**						
Rucaparib and Ipatasertib	Not yet recruiting	Non-Randomized	No	54	Ib	NCT03840200
**PARPi + androgens**						
Olaparib and Testosterone Enanthate or Cypionate	Recruiting		Yes	30	II	NCT03516812
**PARPi + ATR inhibitors**						
Olaparib and AZD6738	Not yet recruiting	Non-Randomized	No	47	II	NCT03787680
**PARPi + GnRH antagonists**						
Olaparib and Degarelix	Recruiting	Randomized	No	20	I	NCT02324998
**PARPi + nanoparticle conjugate**						
Olaparib and CRLX101	Recruiting	Non-Randomized	No	123	I/II	NCT02769962
**Personalized medicine approach**						
SMMART therapy	Not yet recruiting		No	52	I	NCT03878524
**PARPi + radiation treatment**						
Olaparib and RT	Recruiting	Randomized	No	112	I/II	NCT03317392

* Two-stage design study will conduct enrichment of study population before entering stage 2 if the original population has less than desired number of confirmed HRD associated gene aberrations. † Part 1 of the study confirms the starting dose of talazoparib in combination with enzalutamide in genetically unselected population. CTID: Clinical trials identifier; HRD Selection: prescreening of homologous recombination deficiency associated mutations and preselection prior to treatment; RP: Radical prostatectomy; RT: Radiation therapy.Information was compiled by searching the ClinicalTrials.gov. The search was conducted under ‘Condition or disease’ of ‘Prostate cancer’ and additional search terms included ‘Olaparib’, ‘AZD2281’, ‘KU-0059436’, ‘Rucaparib’, ‘Niraparib’, ‘MK-4827’, ‘Talazoparib’, ‘BMN 637’, ‘MDV3800’, ‘Veliparib’, ‘CEP-9722’, ‘Pamiparib’ and ‘BGB-290’.
